# Correction: Cebrián-Ponce et al. Bioelectrical, Anthropometric, and Hematological Analysis to Assess Body Fluids and Muscle Changes in Elite Cyclists during the Giro d’Italia. *Biology* 2023, *12*, 450

**DOI:** 10.3390/biology12060863

**Published:** 2023-06-15

**Authors:** Álex Cebrián-Ponce, Alfredo Irurtia, Jorge Castizo-Olier, Manuel Vicente Garnacho-Castaño, Javier Espasa-Labrador, Zeasseska Noriega, Marta Carrasco-Marginet

**Affiliations:** 1 INEFC-Barcelona Sports Sciences Research Group, Institut Nacional d’Educació Física de Catalunya (INEFC), Universitat de Barcelona (UB), 08038 Barcelona, Spain; acebrian@gencat.cat (Á.C.-P.); airurtia@gencat.cat (A.I.); franciscojavierespasa@gencat.cat (J.E.-L.); znoriega@gencat.cat (Z.N.); 2 School of Health Sciences, TecnoCampus, Pompeu Fabra University, 08302 Barcelona, Spain; jcastizo@tecnocampus.cat; 3 DAFNiS Research Group (Pain, Physical Activity, Nutrition and Health), Campus Docent Sant Joan de Déu, University of Barcelona, 08830 Sant Boi de Llobregat, Spain; mavigarcas@gmail.com; 4 Faculty of Health Sciences, Valencian International University (VIU), 46002 Valencia, Spain

## Error in Table

In the original publication [1], there was a mistake in Table 2 as published. The words “ICW (L)” and “ECW (L)” are inverted. The corrected order appears below. The authors state that the scientific conclusions are unaffected. This correction was approved by the Academic Editor. The original publication has also been updated.

## Figures and Tables

**Table 2:** Hematological, anthropometric, and bioelectrical changes of elite cyclists during the Giro d’Italia 2013.

				PRE-MID		MID-POST		PRE-POST	
	PRE	MID	POST	∆ (%)	*p*	∆ (%)	*p*	∆ (%)	*p*
Body composition									
BM (kg)	70.5 ± 6.1	70.4 ± 6.2	70.7 ± 5.9	−0.1 ± 1.2	0.574	0.4 ± 1.3	0.400	0.3 ± 1.6	0.499
BMI (kg/m^2^)	21.4 ± 0.8	21.4 ± 0.8	21.4 ± 0.7	−0.1 ± 1.2	0.589	0.4 ± 1.3	0.443	0.3 ± 1.6	0.673
TBW (L)	45.9 ± 3.7	46.1 ± 4.0	45.5 ± 3.3	0.3 ± 1.6	0.398	−1.2 ± 2.1	0.075	−1.0 ± 1.5	0.086
ECW (L)	15.3 ± 1.2	15.3 ± 1.2	15.1 ± 1.0	0.3 ± 1.7	0.683	−1.3 ± 2.2	0.105	−1.1 ± 1.6	0.064
ICW (L)	30.7 ± 2.5	30.8 ± 2.8	30.3 ± 2.2	0.3 ± 1.4	0.458	−1.0 ± 1.9	0.090	−0.8 ± 1.4	0.079
ECW/ICW ratio	0.50 ± 0.01	0.50 ± 0.01	0.50 ± 0.00	−0.1 ± 0.3	1.000	0.3 ± 0.3	0.083	0.2 ± 0.3	0.083
Whole-body BIVA									
R/H (Ω/m)	265.5 ± 21.5	262.8 ± 24.4	275.9 ± 13.9	−1.0 ± 3.7	0.515	5.4 ± 5.9	0.008 *	4.2 ± 4.6	0.038
Xc/H (Ω/m)	35.8 ± 2.8	34.3 ± 3.7	37.5 ± 2.2	−4.3 ± 6.8	0.108	10.5 ± 11.8	0.013 *	5.1 ± 4.6	0.018
Z/H (Ω/m)	267.9 ± 21.6	265.0 ± 24.6	278.4 ± 13.9	−1.1 ± 3.8	0.515	5.5 ± 6.0	0.008 *	4.2 ± 4.6	0.038
PhA (°)	7.7 ± 0.4	7.4 ± 0.4	7.8 ± 0.4	−3.4 ± 4.0	0.048 *	4.7 ± 7.7	0.089	1.0 ± 4.9	0.623
Mean quadriceps BIVA									
R/H (Ω/m)	14.7 ± 1.3	15.3 ± 1.3	15.1 ± 2.4	4.6 ± 10.6	0.236	−1.4 ± 13.6	1.000	2.8 ± 15.9	0.624
Xc/H (Ω/m)	5.1 ± 0.5	5.2 ± 0.5	5.4 ± 0.5	1.3 ± 12.2	0.671	5.8 ± 12.5	0.256	6.4 ± 11.2	0.122
Z/H (Ω/m)	15.6 ± 1.4	16.1 ± 1.3	16.0 ± 2.4	4.2 ± 10.4	0.236	−0.6 ± 13.1	0.722	3.2 ± 14.9	0.553
PhA (°)	19.4 ± 1.6	18.7 ± 1.3	20.1 ± 2.0	−3.1 ± 8.4	0.314	7.7 ± 10.3	0.085	4.2 ± 11.9	0.477
Mean hamstrings BIVA									
R/H (Ω/m)	14.5 ± 2.0	14.9 ± 1.1	14.6 ± 1.6	3.9 ± 13.7	0.463	−1.4 ± 9.4	0.766	2.0 ± 13.1	0.675
Xc/H (Ω/m)	4.9 ± 0.4	4.9 ± 0.5	5.0 ± 0.9	−1.1 ± 9.3	0.608	3.3 ± 13.0	0.440	2.3 ± 17.0	0.866
Z/H (Ω/m)	15.3 ± 2.0	15.6 ± 1.1	15.5 ± 1.7	3.3 ± 12.6	0.574	−0.9 ± 9.6	0.953	2.0 ± 13.2	0.722
PhA (°)	18.9 ± 1.9	18.1 ± 2.0	18.9 ± 2.1	−3.9 ± 9.3	0.262	4.2 ± 6.1	0.050 *	0.0 ± 8.8	0.953
Mean calves BIVA									
R/H (Ω/m)	36.2 ± 3.3	33.1 ± 3.3	34.7 ± 3.4	−8.7 ± 3.2	0.008 *	5.0 ± 4.5	0.017 *	−4.2 ± 3.7	0.018 *
Xc/H (Ω/m)	8.6 ± 1.2	7.6 ± 1.2	8.5 ± 1.2	−10.9 ± 7.3	0.007 *	11.5 ± 12.7	0.028 *	−1.1 ± 9.8	0.357
Z/H (Ω/m)	37.2 ± 3.4	33.9 ± 3.4	35.7 ± 3.6	−8.8 ± 3.3	0.008 *	5.3 ± 4.7	0.013 *	−4.1 ± 3.7	0.017 *
PhA (°)	13.3 ± 1.1	13.0 ± 1.2	13.7 ± 1.0	−2.5 ± 5.8	0.286	6.0 ± 9.2	0.097	3.1 ± 8.7	0.498
Hematological									
Hb (g/dL)	14.1 ± 0.7	13.1 ± 0.9	13.6 ± 0.8	−7.3 ± 3.5	0.008 *	4.1 ± 3.0	0.007 *	−3.6 ± 2.6	0.012 *
Hct (%)	42.0 ± 2.3	39.3 ± 2.3	40.6 ± 2.2	−6.4 ± 2.7	0.008 *	3.6 ± 3.5	0.015 *	−3.1 ± 3.3	0.028 *
EPV (mL)	2663.3 ± 280.2	2785.4 ± 309.4	2730.2 ± 257.9	13.1 ± 6.2	0.011 *	−5.9 ± 4.5	0.050 *	6.2 ± 5.4	0.028 *
POsm (mOsm/L)	285.4 ± 6.2	292.2 ± 16.9	312.2 ± 33.1	2.4 ± 5.5	0.362	7.2 ± 13.0	0.155	9.3 ± 10.8	0.011 *
Na^+^ (mEq/L)	138.7 ± 1.9	141.0 ± 1.4	143.4 ± 6.0	1.7 ± 1.6	0.027 *	1.7 ± 4.0	0.233	3.5 ± 4.7	0.049 *
K^+^ (mEq/L)	5.1 ± 0.2	4.7 ± 0.3	4.8 ± 0.3	−9.3 ± 5.9	0.012 *	3.2 ± 7.3	0.396	−6.7 ± 5.8	0.017 *

BIVA, bioelectrical impedance vector analysis; BM, body mass; BMI, body mass index; ECW, extracellular water; EPV, estimated plasma volume; Hb, hemoglobin concentration; Hct, hematocrit; ICW, intracellular water; K^+^, plasma potassium concentration; MID, second assessment at the middle of the race; Na^+^, plasma sodium concentration; PhA, phase angle; PRE, first assessment one day before the start of the race; POsm, plasma osmolality; POST, last assessment at the end of the race; R/H, height-adjusted resistance; TBW, total body water; Xc/H, height-adjusted reactance; Z/H, height-adjusted impedance; Δ, delta value (%); * *p* statistical significance, *p* < 0.05.
